# *(R)*-[^11^C]Verapamil PET studies to assess changes in P-glycoprotein expression and functionality in rat blood-brain barrier after exposure to kainate-induced status epilepticus

**DOI:** 10.1186/1471-2342-11-1

**Published:** 2011-01-03

**Authors:** Stina Syvänen, Gert Luurtsema, Carla FM Molthoff, Albert D Windhorst, Marc C Huisman, Adriaan A Lammertsma, Rob A Voskuyl, Elizabeth C de Lange

**Affiliations:** 1 Division of Pharmacology, LACDR, Leiden University, P.O. Box 9502, 2300 RA Leiden, The Netherlands; 2 Department of Nuclear Medicine & Molecular Imaging, Groningen University Medical Center, P.O. Box 30.001 9700 RB Groningen, The Netherlands; 3 Department of Nuclear Medicine & PET Research, VU University Medical Center, P.O. Box 7057, 1007 MB, Amsterdam, The Netherlands; 4 Epilepsy Institute of The Netherlands Foundation (SEIN), P.O. Box 21, 2100 AA, Heemstede, The Netherlands

## Abstract

**Background:**

Increased functionality of efflux transporters at the blood-brain barrier may contribute to decreased drug concentrations at the target site in CNS diseases like epilepsy. In the rat, pharmacoresistant epilepsy can be mimicked by inducing status epilepticus by intraperitoneal injection of kainate, which leads to development of spontaneous seizures after 3 weeks to 3 months. The aim of this study was to investigate potential changes in P-glycoprotein (P-gp) expression and functionality at an early stage after induction of status epilepticus by kainate.

**Methods:**

*(R)*-[^11^C]verapamil, which is currently the most frequently used positron emission tomography (PET) ligand for determining P-gp functionality at the blood-brain barrier, was used in kainate and saline (control) treated rats, at 7 days after treatment. To investigate the effect of P-gp on *(R)*-[^11^C]verapamil brain distribution, both groups were studied without or with co-administration of the P-gp inhibitor tariquidar. P-gp expression was determined using immunohistochemistry in post mortem brains. *(R)*-[^11^C]verapamil kinetics were analyzed with approaches common in PET research (Logan analysis, and compartmental modelling of individual profiles) as well as by population mixed effects modelling (NONMEM).

**Results:**

All data analysis approaches indicated only modest differences in brain distribution of *(R)*-[^11^C]verapamil between saline and kainate treated rats, while tariquidar treatment in both groups resulted in a more than 10-fold increase. NONMEM provided most precise parameter estimates. P-gp expression was found to be similar for kainate and saline treated rats.

**Conclusions:**

P-gp expression and functionality does not seem to change at early stage after induction of anticipated pharmacoresistant epilepsy by kainate.

## Background

About 30-40% of all people with epilepsy do not become fully seizure free with present medication, even when treated at the maximal tolerated dose. This pharmacoresistance is particularly prominent in partial epilepsies and some severe syndromes in infants, but essentially it can occur in nearly all types of epilepsies and epileptic syndromes. In addition, unresponsiveness in these patients is not limited to a specific drug or drug class, but occurs with the complete range of antiepileptic drugs (AEDs) [1,2].

For a drug to exert its effect, it has to be distributed to its target at a therapeutic concentration and must be able to interact with the target. Several different mechanisms influence transport across the blood-brain barrier (BBB); i.e. passive diffusion, as well as active influx and active efflux processes. P-glycoprotein (P-gp) is one of the most important efflux transporters of exogenous substances at the BBB [3,4]. Several studies, both clinical and pre-clinical, have indicated that P-gp functionality at the BBB may contribute to decreased target site AED concentrations in the brain [5-15]

A number of studies have shown that BBB P-gp expression is upregulated in epilepsy [10,12,16-20]. Therefore, it had been hypothesized that the observed loss of efficacy of AEDs may be caused by limited brain distribution in pharmacoresistant epilepsy caused by P-gp mediated efflux from the brain [21]. This hypothesis has been criticised by some authors [22].

Temporal Lobe Epilepsy is the most common form of epilepsy in humans and in more than half of the patients it is associated with pharmacoresistance. This condition can be mimicked in the rat by inducing status epilepticus (SE), e.g. by intraperitoneal injection of kainate [23] . In the majority of animals this leads to development of spontaneous seizures after about three weeks. However as not all patients and not all SE subjected rats become pharmacoresistant it is an important question whether this variability is related to the degree of increased P-gp expression and whether this parameter can be used as a predictor for development of pharmacoresistance.

The aim of this study was to investigate potential changes in P-gp expression and functionality in vivo with positron emission tomography (PET). For this purpose a condition at which there is clear increased P-gp expression would be most suitable. In SE models the most prominent increase in P-gp expression has been reported between 2-7 days after SE induction [6,20,24]. The well established PET ligand for determining P-gp functionality at the BBB, *(R)*-[^11^C]verapamil [25-34], was used in kainate and saline treated rats, at 7 days after injection. To investigate the specific contribution of P-gp at the BBB, both groups were studied without or with co-administration of the P-gp inhibitor tariquidar. P-gp expression was further determined using immunohistochemistry in post mortem brains to investigate the relationship between expression and functionality. *(R)*-[^11^C]verapamil kinetics in these groups were analyzed using the common data analysis approaches used in PET (Logan analysis, compartmental modelling of individual profiles) as well as by population mixed effects modelling (NONMEM) in which sources of variability can be better addressed. In addition, covariate analysis can be integrated in the NONMEM data analysis which is a valuable tool to identify the specific origin of variability when different animal types and treatments are used.

## Methods

### Animals

Adult male Sprague-Dawley rats (n = 50, Harlan, Horst, The Netherlands) weighing 200-224 g at arrival were housed in groups of 5-6 per cage until treatment. They were kept at a constant temperature of 21°C and at a 12 h light/dark cycle, in which lights were switched on at 8:00 AM. Animals had unrestricted access to food (Teklad Global 16% Protein Rodent Diet, Harlan, Madison, WI, USA) and water. Animal procedures were performed in accordance with Dutch laws on animal experimentation. All experiments were approved by the Ethics Committee for Animal Experiments of Leiden University (approval number UDEC08178). After approximately one week of habituation and 7 days prior to PET investigation rats were treated with either kainic acid (5 mg/mL) or saline. Kainic acid was administered repeatedly as a 10 mg/kg (2 mL/kg) i.p. injection followed by 5 mg/kg (1 mL/kg) i.p. every 30-60 minutes until stage IV seizures according to Racine's scale [35] occurred, or when a total amount of 30 mg/kg kainic acid was reached. Control rats were treated with 3 saline i.p. injections using the same volumes as for the kainate treated rats, 30 minutes apart. After treatment rats were housed individually.

### Chemicals

Tariquidar was obtained from API Services Inc (Westford, MA, USA), kainic acid and Triton X-100 from Sigma-Aldrich (Zwijndrecht, The Netherlands), isoflurane from Pharmachemie BV (Haarlem, The Netherlands), 5% glucose in saline from hospital pharmacy of the University Medical Centre Leiden (UMCL, Leiden, The Netherlands), Tissue-Tek from Sakura Finitek Europe (Zoeterwoude, The Netherlands), iso-methylbutane and P-gp primary antibody C219 from VWR (Amsterdam, The Netherlands), bovine serum albumin from ICN Biomedicals (Zoetermeer, The Netherlands), rabbit and swine serum from Tebu-Bio (Heerhugowaard, The Netherlands), secondary antibody (Rabbit-anti-Mouse, bRaM) from Dako (Heverlee, Belgium) and streptavidin and 3,3-diaminobenzidine (DAB)-kit from Brunschwig Chemie Vector Labs (Amsterdam, The Netherlands). All starting materials for *(R)*-[^11^C]verapamil syntheses were obtained commercially and were of reagent grade.

### PET experiments

Rats were anesthetized via a nose mask with isoflurane, 4% and 2% induction and maintenance, respectively, in oxygen 1 L/minute. The femoral vein and artery were cannulated 1-2 hours prior to PET scanning for administration of *(R)*-[^11^C]verapamil and tariquidar, and blood sampling, respectively.

*(R)*-[^11^C]verapamil was synthesized as previously described [36] yielding a solution of 1-3 GBq in around 10 mL 10% ethanol in saline. The specific activity was 56 ± 26 (mean ± s.d.) GBq/μmole. Rats from the different groups were randomly assigned to experimental days and scanned in pairs for 60 minutes using a double LSO/LYSO layer High Resolution Research Tomograph (HRRT, CTI/Siemens, Knoxville, TN, USA) [37]. For attenuation and scatter correction, transmission scans were acquired prior to the start of the emission scan using a 740 MBq 2-dimensional (2D) fan-collimated ^137^Cs (662 keV) moving point source [38]. The emission scan was started 30 seconds before an injection of 20.8 ± 4.5 MBq (0.15-0.3 mL) no-carrier added *(R)*-[^11^C]verapamil (Table 1). Half of the rats were injected intravenously with tariquidar, 15 mg/kg (3 mL/kg) dissolved in a vehicle consisting of 5% glucose in saline, as a bolus dose 20-30 minutes prior to the *(R)*-[^11^C]verapamil injection. The other half was treated only with vehicle (3 mL/kg). Immediately after the *(R)*-[^11^C]verapamil scan, 8.9 ± 1.8 MBq (0.15-0.3 mL) [^18^F]-2-fluoro-2-deoxy-D-glucose (^18^FDG) was injected and a new 60 minutes scan was started. The purpose of the ^18^FDG scan was to outline the brain to aid in defining regions of interest. The HRRT scanner acquires data in list mode, enabling post-scan definition of the frame sequence. The final frame sequence used for analysis was 6 × 10, 2 × 30, 3 × 60, 2 × 150, 2 × 300 and 4 × 600 seconds. Studies were reconstructed using an iterative 3 D ordered-subsets weighted least-squares (3D-OSWLS) method with 8 iterations and 16 subsets [39]. No post-reconstruction filtering was applied. Point source resolution varied across the field of view from approximately 2.3 to 3.2 mm (FWHM) in the transaxial direction and from 2.5 to 3.4 mm in the axial direction [37].

**Table 1:** Number of animals (n) with weight at the time of PET scanning and injected doses of (R)-[11C]verapamil and 18FDG in the different treatment groups (mean ± s.d).

	Saline and vehicle treated	Kainate and vehicle treated	Saline and tariquidar treated	Kainate and tariquidar treated	All groups
n	10	11	10	11	42
Body weight (g)	327 ± 26	292 ± 33	326 ± 25	293 ± 24	308 ± 31
*R*-[^11^C]verapamil (MBq)	22.3 ± 3.9	21.4 ± 4.8	22.1 ± 3.9	19.4 ± 5.1	20.8 ± 4.5
^18^FDG (MBq)	9.6 ± 2.1	8.5 ± 1.9	9.3 ± 1.6	9.1 ± 1.7	8.9 ± 1.8

Of the original 50 animals, 8 animals were discarded; 4 died as a result from the kainate treatment, 1 did not respond to kainate treatment, and 3 were excluded for technical reasons, such as movement in the scanner or extra vascular tracer injection.

During the *(R)*-[^11^C]verapamil scan, arterial blood samples of 0.1 mL were obtained at 0.5, 1, 3, 5, 10, 15, 20, 30, 45 and 60 minutes after injection. Plasma was obtained by centrifugation at 5000 rpm for 5 minutes and activity in plasma and whole blood samples was measured in an automated 1282 Compugamma CS Universal Gamma Counter (LKB Wallac, Turku, Finland), cross-calibrated against the PET scanner. In addition, blood samples of 0.3 mL were obtained at 10, 30 and 60 minutes and analysed for metabolism of *(R)*-[^11^C]verapamil. A further 0.3 mL blood sample for the measurement of tariquidar concentration was obtained after the end of the ^18^FDG scan and analyzed using liquid chromatography tandem mass-spectrometry (LC/MS). Rats were euthanized under anaesthesia by decapitation immediately after the final blood sample.

### Metabolite analysis

Metabolite analysis in plasma was performed as described previously [40]. In short, using solid phase extraction, three fractions were determined for each plasma sample: *(R)-*[^11^C]verapamil, N-dealkylated products and O-demethylated products (non-polar metabolite fraction) and N-demethylated products (polar metabolite fraction).

### P-glycoprotein expression

P-gp expression in brain capillaries was measured according to a previously published protocol after some minor changes [41]. In short, after decapitation, the brain was isolated and transferred to an aluminium foil cup filled with Tissue-Tek, embedding the brain, and placed in an iso-methylbutane containing glass beaker in liquid nitrogen. After 1-2 minutes the brain was completely frozen and thereafter stored at -80°C until further analysis. The frozen brains were cut in coronal sections, 30 μm, using a Leica CM1900 cryostat (Leica Microsystems B.V., Rijswijk, The Netherlands). The sections were post fixed in acetone and stored at -20°C until immunohistological staining. Sections selected from the brain between -2.3 mm to bregma, were chosen for immunohistochemistry [42].

Staining of brain sections from all rats were processed simultaneously in the same reaction trays to obtain comparable staining intensity. Sections were thoroughly washed for 3 × 5 minutes in 0.5 M tris-buffered saline (TBS, 9% NaCl, pH 7.6) at room temperature. To inhibit endogenous peroxidase and reduce background staining, sections were incubated for 30 minutes in 0.5% H_2_O_2 _in TBS. Again, sections were washed 3 × 5 minutes in 0.5 M TBS. To block interaction with other substrates, a solution containing 3% bovine serum albumin (BSA), 11% normal rabbit serum and 0.3% Triton X-100, was added and incubated for 60 minutes at room temperature. After this, sections were incubated overnight at 4°C in 1:100 monoclonal mouse primary antibody C219 in carrier medium (containing 1% BSA, 1% rabbit serum and 0.3% Triton X-100 in TBS). After overnight incubation, sections were washed 3 × 10 minutes in 0.5 M TBS at room temperature. Next, sections were incubated for 90 minutes in 1:200 biotin-conjugate Rabbit-anti-Mouse (bRaM) secondary antibody in the same carrier solution as the primary antibody mentioned above. Sections were washed 3 × 5 minutes in 0.5 M TBS and incubated for 90 minutes in 1:375 in 0.5 M TBS solution, after which they were washed 3 × 5 minutes in 0.5 M TB (pH 7.6) instead of TBS to avoid possible interaction between saline and 3,3-diaminobenzidine (DAB). Sections were incubated for 10 minutes in DAB with 0.03% H_2_O_2 _and then washed 2 × 5 minutes in 0.5 M TB and 1× in ultrapurified water (ELGA, Ede, The Netherlands). Slides were air-dried and, on the next day, cover slipped with Entallan (Merck, Darmstadt, Germany).

Images of the stained sections were obtained with a Leica DM 600B microscope (Leica Microsystems). All analyses were performed using the freely available software Image J [43]. Two measurements, the P-gp labelled surface area and its optical density, were performed on two sections per animal and region in the regions of the cortex and hippocampus CA1. Both the labelled surface area and optical density value was obtained by first choosing a global threshold value, used for all slides. Only grey values above this threshold were assumed to result from P-gp staining. In the area method, the total number of pixels above the threshold was summed. For the optical density, the mean grey value was measured for each slide and the background, specific for that particular slide, was subtracted from the mean value.

### Data analysis

PET image data were analyzed using the freely available software package Amide 0.8.22 [44]. Time-activity curves in whole brain where obtained by drawing a volume of interest (VOI) in 4 adjacent transaxial planes of the ^18^FDG images summed over 60 minutes, including cortical and central structures. The final VOI volume was 127 μL. Other VOIs were drawn in cerebellum (3 planes) and liver (3 planes), resulting in VOI volumes of 49 and 57 μL, respectively. Next, VOIs were transferred to the dynamic *(R)*-[^11^C]verapamil image sequence, generating corresponding time-activity curves. Whole brain and cerebellum time-activity curves were used for analysis of *(R)*-[^11^C]verapamil kinetics in brain. To correct for an intravascular contribution, radioactivity measured in whole blood was exponentially interpolated to obtain blood radioactivity values at each mid frame time point and then subtracted from the brain time-activity curves, assuming that cerebral blood volume was 3% of the total brain volume [45].

Liver time-activity curves, obtained from the PET images, and plasma time-activity curves, obtained from the plasma samples, were checked for any irregularities pointing to e.g. extravascular injection. If irregularities were found, the animal was excluded from further analysis.

Plasma time-activity curves were corrected for tracer metabolism before they were used as input for kinetic modelling. As metabolite fractions were not measured at each plasma time point, linear regression was used to estimate the fraction of intact *(R)*-[^11^C]verapamil at all blood sampling time points. The activity in each plasma sample was then multiplied with the fraction of intact *(R)*-[^11^C]verapamil. Two types of corrections were used: (1) complete metabolite correction and (2) correction only for polar metabolites. The rationale for the second correction was that non-polar metabolites of *(R)*-[^11^C]verapamil are thought to have similar kinetics as *(R)*-[^11^C]verapamil itself [46]. Mathematically, this is described by the following equations:

(1) $$C_{p}=C_{m}*f$$

where *C_p _*is the concentration of the plasma input function as used for modelling purposes, *C_m _*the (total) plasma concentration as measured in the gamma counter and *f *the fraction of unchanged *(R)*-[^11^C]verapamil. Here, *f *is a value between 0 and 1, given by:

(2a) $$f=\frac{{\left(R\right)}^{\_}{\text{[}}^{\text{11}}\text{C]verapamil activity }}{\text{Total plasma activity}}$$

for complete metabolite correction, or

(2b) $$\begin{array}{l} f=\frac{{\left(R\right)}^{\_}{\text{[}}^{\text{11}}\text{C]verapamil activity}}{\text{Total plasma activity}}\\ +\frac{{\text{[}}^{\text{11}}\text{C]non - polar metabolite activity}}{\text{Total plasma activity}} \end{array}$$

for correction of polar metabolites only

Finally, data were analysed without a correction for labelled metabolites, i.e. assuming *f = 1*. For modelling of individual profiles (see section below), C_p _was linearly interpolated to obtain plasma activity values at the same time points as in the brain, i.e. the mid frame time points.

#### Modelling of individual profiles

The models described in this section were fitted to individual data from each animal. To obtain a model-independent estimate of the brain-to-blood partition coefficient K_p _(often referred to as volume of distribution, V_T, _in PET literature), Logan graphical analysis [47] was used. In addition, the influx rate constant K_1 _was estimated from Logan analysis. PET data were also fitted using standard compartmental models, i.e. a 1 tissue (brain) compartment model with 2 rate constants (1T2k) and a 2 tissue (brain) compartment model with 4 rate constants (2T4k) [48]. In the 1T2k model the two rate constants describe transport across the BBB, whilst the 2T4k model also includes two rate constants describing the exchange between fast and slow equilibrating compartments in the brain. The model with the lowest value of the Akaike information criterion (AIC) was considered the best [49]. Rate constants obtained for the different rat groups were compared using a t-test with Bonferroni correction for multiple comparisons. Dedicated analysis programs were written within the MATLAB 6 (Mathworks, Natick, MA, USA) software environment.

#### Population mixed effects modelling

Nonlinear mixed effects modelling, using NONMEM VI (GloboMax LLC, Hanover, MD, USA), provide a tool for analyzing repeated measurements data in which the relationship between the explanatory and response variables can be modeled as a single function, allowing the parameters to differ between individuals. In addition, these techniques recognize that the variability associated with the response variable for a given individual may depend on the response value in a way that is similar for all individuals. This could be due, for example, to properties associated with measurement error.

Data from all rats was processed simultaneously. The subroutine ADVAN 9 and first-order conditional estimation with interaction were used throughout the modelling procedure. Model selection was based on the objective function value (OFV; with the lowest value corresponding to the best model), model parameter uncertainty and visual analysis using software Xpose 4 [50] implemented in R 2.7.1 (The R foundation for Statistical Computing) accessed from Census [51]. For nested models, OFV reductions of 3.83, 6.63 and 10.83 units correspond to improved fits at p < 0.05, p < 0.01 and p < 0.001 levels, respectively. The inter-individual variation of a parameter was described by the exponential variance model:

(3) $$\theta_{i}=\theta_{pop}*\exp(\eta_{i})$$

where θ*_i _*is the parameter in the i^th ^animal, θ*_pop _*the parameter in a typical animal and *η*_*i *_the inter-animal variability, which is assumed to be normally distributed around zero with a standard deviation ω. Equation (3) provides a means to distinguish the parameter value for the i^th ^animal from the typical value predicted from the regression model.

The NONMEM model was constructed in two steps. In the first step, a pharmacokinetic model for *(R)*-[^11^C]verapamil plasma concentrations was developed. One, two and three compartment models were evaluated. Treatment (tariquidar or vehicle), rat group (saline or kainate treated) and animal weight were defined as covariates to study their effects on the parameter estimates. In the second step the model was extended to include brain *(R)*-[^11^C]verapamil PET data, while allowing the plasma estimates to freely change from the values obtained in the first step. Again, one and two compartment models were evaluated, and treatment, rat group and animal weight were included as covariates. A stepwise forward addition and backward deletion approach was applied to test the significance for covariate inclusion. Proportional error models were included for the residual variability.

## Results

### Kainate treatment

Kainic acid treatment resulted in stage IV-V seizures in all rats, except one who did not show any epileptic behaviour after 30 mg/kg kainic acid. Typically 3 injections, i.e. 20 mg/kg, were needed and SE was usually reached within 20 minutes after the last injection. Rats usually displayed seizures for 1-2 hours. The seizures were not interrupted by any anti-epileptic drug. Due to the design of this study it was not possible to determine whether these animals eventually would have developed spontaneous epilepsy, but the signs and symptoms during and after SE were very similar to those observed in previous studies where animals did develop epilepsy. Four rats died as a result of kainic acid treatment. The final numbers of successfully scanned rats in each group, together with their average weight on the experimental day, are shown in Table 1.

### (R)-[^11^C]verapamil metabolite analysis

Metabolite analysis showed no differences between plasma metabolite fractions in the various groups of rats. The fractions of intact *(R)*-[^11^C]verapamil were (mean ± s.d.) 0.79 ± 0.05, 0.58 ± 0.07 and 0.39 ± 0.08 at 10, 30 and 60 minutes, respectively. The summed fractions of intact *(R)*-[^11^C]verapamil and its ^11^C labelled non-polar labelled metabolites were 1.00 ± 0.00, 0.97 ± 0.02, 0.91 ± 0.06 at 10, 30 and 60 minutes, respectively. For tariquidar treated rats, complete metabolite correction of the plasma curve (Material and methods, Equation 2a) resulted in better fits and less variation in parameter estimates than either a correction for polar metabolite alone (Equation 2b) or no correction at all. For vehicle rats, the different corrections resulted in only minor differences. In the final analysis complete metabolite correction (Equation 2a) was used and all estimates reported are based on this correction.

### (R)-[^11^C]verapamil brain uptake without tariquidar treatment

**Figure 1:**
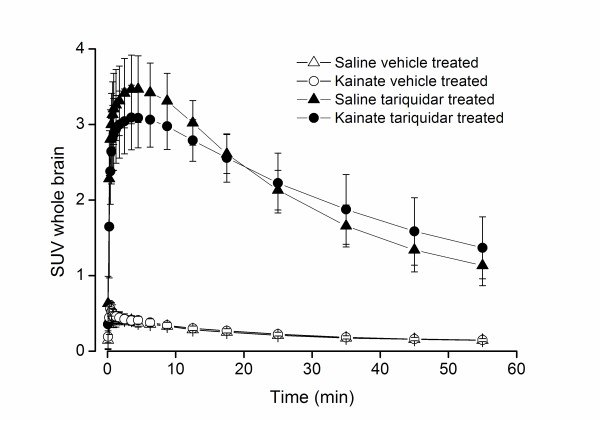
**Mean whole brain uptake of *(R)*-[^11^C]verapamil, expressed as SUV (radioactivity normalized to injected dose and rat weight), as function of time in the four rat groups**. Vertical bars represent standard deviation. No difference was found in the profiles between vehicle co-administered saline and kainate treated animals. Somewhat slower kinetics was observed in the kainate treated compared to the saline treated rats that were also co-administered with tariquidar.

Uptake of *(R)*-[^11^C]verapamil in the brain was homogeneous, but time-activity curves for the cerebellum VOI showed somewhat faster uptake and washout than those for the whole brain VOI (data not shown). Modelling was performed using the whole brain VOI time-activity curves. Average whole brain time-activity curves, expressed in standardized uptake value (SUV, radioactivity normalized to injected dose and animal weight), are shown in Figure 1. Three of the scanned rats were excluded due to movement in the scanner (n = 1) or extravascular tracer injection (n = 2).

**Figure 2:**
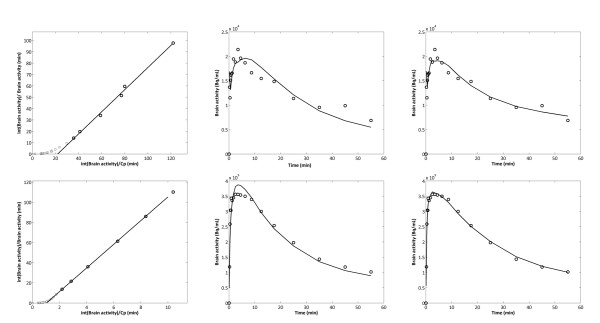
**Logan (left), 1T2k (middle) and 2T4k (right) fits for a vehicle (upper panel) and a tariquidar (lower panel) co-administered saline treated rat**. Circles represent measured concentrations, solid lines best fits. The 2T4k model resulted in better fits than the 1T2k model for most of the rats.

No difference in brain to blood concentration ratio, K_p_, was found between saline treated control and kainate treated rats. All modelling approaches resulted in similar estimates of K_p_, with the 1T2k model providing lower K_p _estimates than Logan analysis, the 2T4k model and the two compartment population model (Table 2). The different modelling approaches (see Material and Methods) also resulted in similar K_1 _estimates with no significant differences between saline and kainate treated rats. AIC values were used to compare the performance of the 1T2k and 2T4k models. In general, AIC values were similar for 1T2k and 2T4k models, with an average difference of 0.5% in favour of the 1T2k model. Visual inspection, however, indicated that, in most cases, better fits were obtained using the 2T4k model (Figure 2). Inter-animal variation in the parameter estimates, however, was larger for the 2T4k model than for the 1T2k model.

**Table 2:** Mean (± standard deviation) of (R)-[11C]verapamil parameter estimates for the different rat groups obtained using various modelling approaches (*** p < 0.001, ** p < 0.01, * p < 0.05., not significant (ns) p> 0.05)

	Vehicle treated			Tariquidar treated				
Parameter estimates	Group 1: Saline	Group 2: Kainate	Group 1 vs Group 2	Group 3: Saline	Group 4: Kainate	Group 3 vs Group 4	Group 1 vs Group 3	Group 2 vs Group 4
Model - Logan								
K_1 _(mL· mL^-1· ^min^-1^)	0.06 (0.02)	0.06 (0.02)	ns	0.85 (0.16)	0.74 (0.18)	ns	***	***
K_p _(mL· mL^-1^)	1.04 (0.19)	1.00 (0.13)	ns	10. 9 (1.3)	11.7 (1.3)	ns	***	***
								
Model - 1T2K								
K_1 _(mL· mL^-1· ^min^-1^)	0.06 (0.03)	0.07 (0.03)	ns	0.93 (0.19)	0.66 (0.18)	***	***	***
k_2 _(min^-1^)	0.08 (0.04)	0.08 (0.04)	ns	0.09 (0.02)	0.06 (0.02)	ns	ns	ns
K_p _(mL· mL^-1^)	0.81 (0.14)	0.82 (0.12)	ns	10.4 (1.0)	10.6 (1.4)	ns	***	***
								
Model -2T4K								
K_1 _(mL· mL^-1· ^min^-1^)	0.07 (0.04)	0.08 (0.04)	ns	1.21(0.30)	0.81 (0.27)	***	***	***
k_2 _(min^-1^)	0.16 (0.15)	0.24 (0.26)	ns	0.73 (0.66)	0.43 (0.35)	ns	*	ns
k_3 _(min^-1^)	0.11 (0.14)	0.19 (0.30)	ns	0.93 (0.75)	0.84 (0.65)	ns	**	*
k_4 _(min^-1^)	1.08 (1.56)	0.49 (0.96)	ns	0.20 (0.05)	1.13 (1.82)	ns	ns	ns
K_p _(mL· mL^-1^)	1.17 (0.65)	1.12 (0.40)	ns	11.0 (1.1)	11.1 (1.5)	ns	***	***
								
Model -2 comp PopPK^a^								
K_1 _(mL· mL^-1· ^min^-1^)	0.10 (0.02)	0.10 (0.02)	ns	0.75 (0.14)	0.62 (0.12)	***	***	***
k_2 _(min^-1^)	0.25 (0.08)	0.25 (0.08)	ns	0.10 (0.04)	0.08 (0.03)	*	***	***
k_3 _(min^-1^)	0.10 (0.03)	0.10 (0.03)	ns	0.04 (0.02)	0.03 (0.01)	ns	***	***
k_4 _(min^-1^)	0.06 (0.02)	0.06 (0.02)	ns	0.06 (0.02)	0.06 (0.02)	ns	ns	ns
K_p _(mL· mL^-1^)	0.97 (0.21)	0.97 (0.21)	ns	11.6 (2.6)	11.6 (2.6)	ns	***	***

^a^rate constants were calculated based on the model estimates shown in Table 3 as follows: K_1 _= Q_in_/V_c_(V_c_/V_br1+2_), k_2 _= Q_out_/V_br1_, k_3 _= Q_br_/V_br1 _and k_4 _= Q_br_/V_br2_.

**Figure 3:**
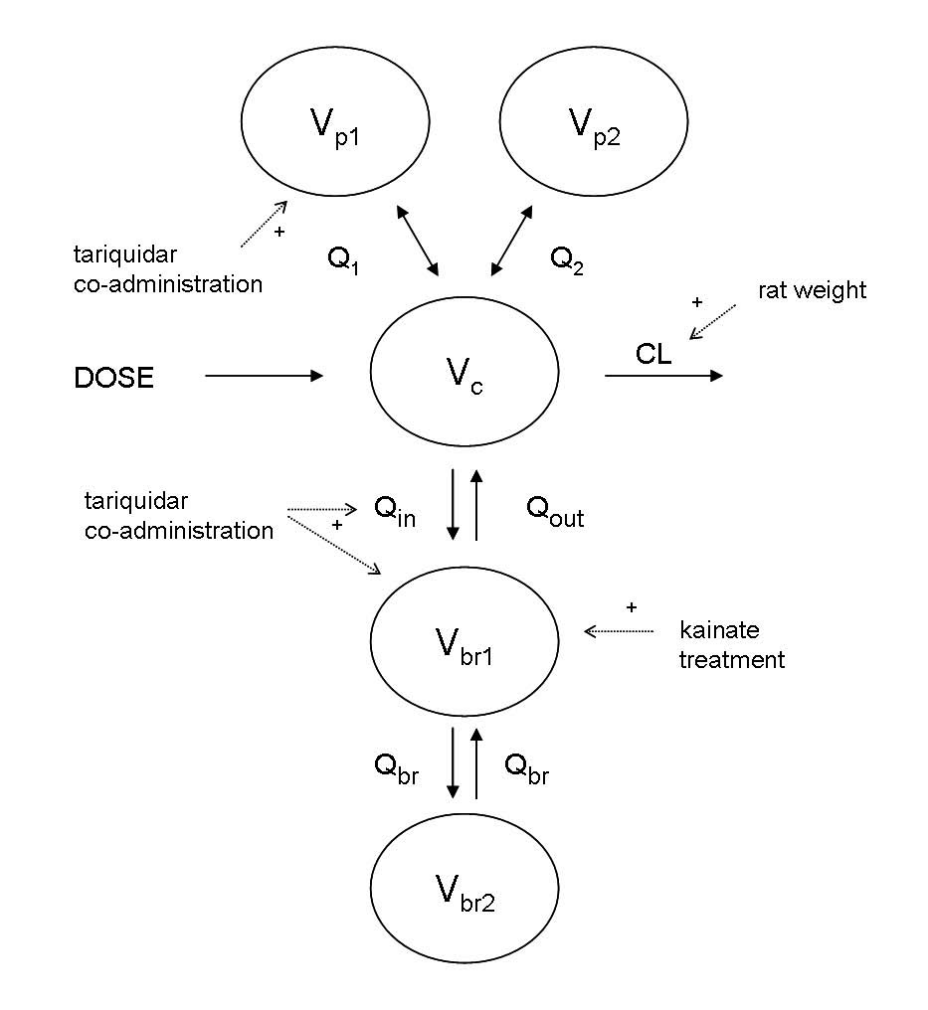
**The final population model**. V_c_, V_p1_, and V_p2 _are pharmacological volumes of distribution in central and two peripheral plasma compartments, respectively. V_br1 _and V_br2 _are volumes of distribution in central and peripheral brain compartment, respectively. CL, Q_1_, Q_2_, Q_in_, Q_out _and Q_br _are total body clearance, bidirectional clearance between plasma and peripheral compartment 1, bidirectional clearance between plasma and peripheral compartment 2, clearance into and out of the brain, and bidirectional clearance between central and peripheral brain compartments respectively. The plus-sign indicates an increase in affected parameter estimate, i.e. tariquidar co-administration increased peripheral plasma volume of distribution (V_p1_), influx clearance to the brain (Q_in_) and brain volume of distribution (V_br1_), animal weight increased systemic clearance (CL), and the kainate treatment increased V_br1_.

The population model is shown in Figure 3. The reader is referred to Gunn, et al. (2001) for a description of the 1T2k and 2T4k models and to Logan, et al. (1990) for a description of the Logan analysis.

### P-gp blockage by Tariquidar co-administration

Tariquidar plasma concentrations were not significantly different between saline and kainate treated rats. At 2 hours after tariquidar administration the concentrations (mean ± s.d.) were 1.8 ± 0.3 and 1.6 ± 0.3 μg/mL in the saline and kainate group, respectively. Tariquidar co-administration resulted in a 10- to 11-fold increase in the brain-to-plasma concentration ratio, K_p_, regardless of the modelling approach used. This increase was of similar magnitude for whole brain and cerebellum. In addition, after tariquidar treatment, there was no significant difference in K_p _between saline and kainate treated rats. Both the 1T2k and the 2T4k model showed that after tariquidar co-administration, the *(R)*-[^11^C]verapamil transport into the brain, K_1 _was strongly increased, both in kainate treated and saline treated rats (Table 2). However K_1 _did not increase to the same extent in both groups, as it was significantly (p < 0.001) lower in kainate compared to saline treated rats. In addition, the transport out of the brain, k_2_, was lower in tariquidar co-administered kainate treated rats compared with the tariquidar co-administered saline treated rats, but the difference did not reach significance. AIC values were lower (2.6%) for the 2T4k model and visual inspection showed that 2T4k fits were better than 1T2k fits (Figure 2).

### Population mixed effects modelling of plasma and brain kinetics with and without tariquidar co-administration

**Figure 4:**
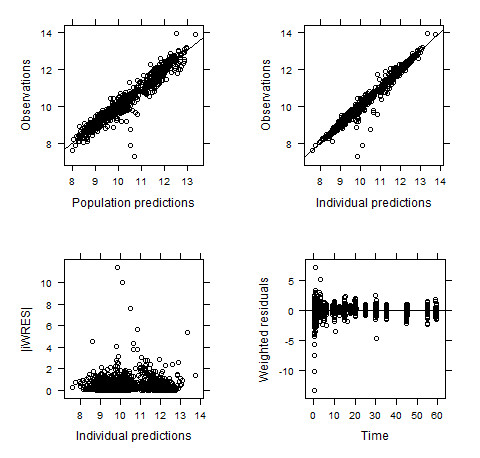
**Diagnostic plots for the final population model including both brain and plasma *(R)*-[^11^C]verapamil concentrations**. All dots represent individual data points and the lines (upper panels) identity lines. Observed versus population and individual predictions are shown in the upper panels, respectively. Most of the data points are randomly distributed around the line of identity which indicates that the model describes the concentrations adequately. Absolute individual weighted residuals versus individual predictions and weighted residuals versus time are shown in the lower panels. Except for some outliers at early time points, most residuals are clustered around zero.

*(R)*-[^11^C]verapamil kinetics in both plasma and brain were further studied using mixed effects modelling by NONMEM. The final model is shown in Figure 3 and the model diagnostics plots are shown in Figure 4. Complete metabolite corrected plasma curves were best described with a three compartment model. There were no significant differences between kainate and saline treated rats (Table 3). Tariquidar co-administration however, resulted in an increased volume of distribution (the pharmacokinetic term) for one of the peripheral compartments (V_p1_), whilst rat body weight affected clearance (CL). Thus, tariquidar co-administration and rat weight were included as covariates for V_p1 _and CL, respectively.

**Table 3:** Population parameter estimates using mixed effects modelling (standard deviation)

Parameter estimates	Estimate	Inter-individual variability	Covariate tariquidar	Covariate kainate	Covariate weight
*Plasma*					
V_c _(mL)	22.8 (2.41)	0.175 (0.085)	-	-	-
V_p1 _(mL)	504 (40.7)		1.20 (0.119)	-	-
V_p2 _(mL)	70.2 (9.91)	0.036 (0.034)	-	-	-
CL (mL· min^-1^)	14.7 (0.681)	0.065 (0.016)	-	-	1.98 (0.362)
Q_1 _(mL· min^-1^)	16.1 (1.75)	0.220 (0.073)	-	-	-
Q_2 _(mL· min^-1^)	22.7 (3.34)	0.220 (0.073)	-	-	-
*Brain*			-		-
V_br1 _(mL)	7.23 (2.00)	0.115 (0.054)	2.41 (0.505)	1.32 (0.416)	-
V_br2 _(mL)	10.7 (2.22)	0.115 (0.054)	-	-	-
Q_in _(mL· min^-1^)	1.75 (0.26)	-	12.0 (0.554)	-	-
Q_out _(mL· min^-1^)	1.81 (0.283)	-	-	-	-
Q_br _(mL· min^-1^)	0.692 (0.143)	-	-	-	-
					
*Residual errors*			-	-	-
blood	0.118 (0.0152)	-	-	-	-
brain	0.226 (0.0354)	-	-	-	-

Covariates for tariquidar co-administration and rat group were defined as θ_covariate_^COVARIATE ^, where the COVARIATEs, i.e. kainate treatment or tariquidar co-administration was 1 and saline treated or vehicle co-administration 0. The numbers reported are thus the fractional differences.

Covariate for weight was defined as (weight/0.3084)^θcovariate ^, where weight represent the individual animal's weight in kg and 0.3084 was the average weight among all animals. See also equations 4 and 5.

Brain kinetics were best described with a two compartment model. Two covariates were included in the brain model: tariquidar co-administration and rat group, i.e. saline or kainate treated. Tariquidar co-administration increased both the transport into the brain, Q_in, _and the volume of distribution in the brain, V_br1_, of *(R)*-[^11^C]verapamil, whilst rat group affected only the V_br1 _estimate. Both tariquidar co-administration and rat group were treated as categorical covariates, i.e. they were assigned a value of 0 or 1. The final equations, including covariates were defined as:

(4) $$\theta_{i}=\theta_{pop}*{\left(weight/308.4\right)}^{\theta_{\mathrm{cov}ariate}}$$

(5) $$\theta_{i}=\theta_{pop}*\theta_{\mathrm{cov}ariate}{}^{COVARIATE\;(1\;or\;0)}$$

Where θ_covariate _is the estimated covariate for weight influence on CL (Equation 4) and for tariquidar co-administration or rat group influence on V_p1_, Q_in _or V_br1 _(Equation 5). In Equation 5, tariquidar co-administration and kainate treatment were assigned the value of 1, while vehicle co-administration and saline treatment were assigned the value of 0. Thus, θ_covariate _is the fractional change caused by kainate treatment or tariquidar co-administration.

Inter-individual variation was investigated for all parameters, but incorporated only for those parameters for which it significantly (ΔOFV > 3.83) improved the model (Table 3). Reparameterisation of CL into rate constants (Table 2) was used to allow for comparison with the other modelling approaches, and showed that tariquidar co-administration increased the rate constant for *(R)*-[^11^C]verapamil transport into the brain, K_1, _and decreased its rate constant for transport out of the brain, k_2, _in both saline and kainate treated rats. In addition, since kainate treated rats had a higher brain distribution volume (V_br1_), k_2 _was reduced in kainate compared to saline treated rats. All these findings were in accordance with those obtained using the other modelling approaches, but the variation in parameter estimates was smaller.

### ^18^FDG brain uptake

At 60 minutes after injection, ^18^FDG brain uptake was significantly decreased in kainate treated rats compared to saline treated rats (p < 0.05), with SUV (mean ± s.d.) of 2.0 ± 0.4 and 1.6 ± 0.3, respectively. No change in ^18^FDG brain uptake could be demonstrated after tariquidar co-administration. SUV was 1.8 ± 0.4 in both groups. The same trend was seen in the cerebellum.

### P-gp expression

**Figure 5:**
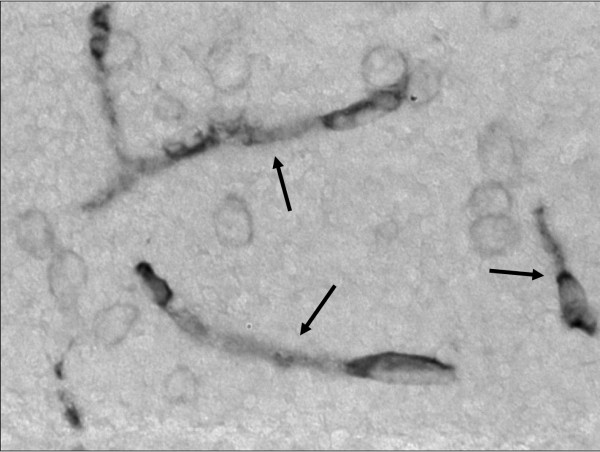
**Staining of P-glycoprotein in the brain capillaries in the region of hippocampus**. The arrows indicate the stained capillaries in a kainate treated animal.

**Figure 6:**
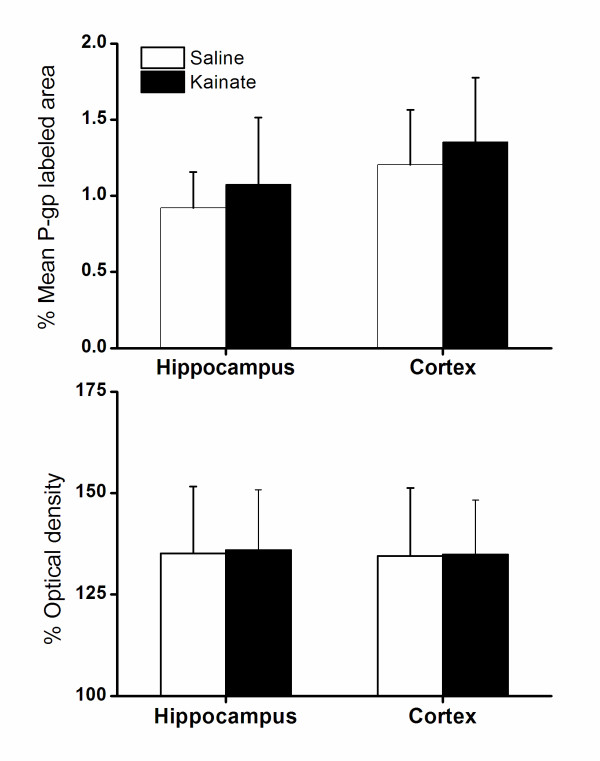
**Mean P-gp expression measured as P-gp labelled area (upper panel) and optical density (lower panel) at 7 days after saline (n = 20) or kainate treatment (n = 22)**. Vertical bars represent standard deviation. There was no significant difference between the two rat groups, but the P-gp labelled area tended to be somewhat larger for kainate treated rats.

P-gp expression was measured immunohistochemically and determined as P-gp labelled surface area and optical density. Results are shown in Figure 5 and 6. The area method showed a trend towards higher P-gp expression in kainate treated rats, although the large variation resulted in a non significant difference when groups were compared with the student's t-test (p > 0.05). The optical density method showed no difference, not even a trend, in P-gp expression between saline and kainate treated rats.

In summary, all data analysis approaches indicated only modest differences in brain distribution of *(R)*-[^11^C]verapamil between saline and kainate treated rats. The most significant difference was in the transport rate of *(R)*-[^11^C]verapamil into the brain (K_1 _and Q_in_). However, this difference was only significant in the tariquidar treated groups. *(R)*-[^11^C]verapamil kinetics were best described with a two compartment plasma and two compartment brain model. Tariquidar co-administration resulted mainly in an increase of *(R)*-[^11^C]verapamil transport into the brain. NONMEM provided most precise parameter estimates. P-gp expression was found to be similar for kainate and saline treated rats.

## Discussion

In this study the P-gp substrate *(R)*-[^11^C]verapamil was used in rats to investigate P-gp function at the BBB at 7 days following saline injection (control) and induction of status epilepticus (SE) by kainate. To the best of our knowledge, this is the first PET study of P-gp function at the BBB of kainate treated rats. In addition, it is also the first study in which PET data were analyzed both on individual data profiles using kinetic models as used routinely by the PET community, and with population mixed effects modelling, as used in the pharmacokinetic and pharmacokinetic-pharmacodynamic research field.

It has been hypothesized that P-gp expression is upregulated in epilepsy and hence contributes to the development of pharmacoresistance [10-12,21,52,53]. In the present study co-administration of *(R)*-[^11^C]verapamil with vehicle showed no or only small BBB transport differences between saline and kainate treated rats. *(R)*-[^11^C]verapamil uptake in the brain was very low in both groups. A small increase in P-gp expression/function and thereby also a small decrease in *(R)*-[^11^C]verapamil uptake in kainate treated rats might not be detectable due to the already low baseline of *(R)*-[^11^C]verapamil in saline treated (control) rats. Further, due to the low baseline signal and scanner resolution the analysis was done on a whole brain region and hence possible regional differences between kainate treated and non-treated rats could therefore not be investigated.

In the present study rats were scanned one week after induction of SE by kainate. This time point was chosen because previous studies indicated that the most pronounced P-gp expression was expected around this time [24]. However, at this time point in the present study, immunohistochemical staining of P-gp expression in the hippocampus revealed only a small trend in kainate treated rats. This was in line with P-gp functionality measured using PET, i.e. no or very small differences between the two groups. Bankstahl et al reported increase in P-gp expression at 2 days post SE induced by pilocarpine and amygdala stimulation but not at 7 days post SE [20]. On the other hand van Vliet et al, who induced SE by electrical stimulation in the dentate gyrus, and by pilocarpine reported prominent increase on both time points [6,24]. These variable results may thus be caused by the use of different animal models and differences experimental conditions. Further, different staining protocols emphasize P-gp expression in different cell types in the brain [41]. The immunostaining used in this study shows P-gp expression in the endothelial cells of the BBB.

Previous studies using the kainate model have shown that spontaneous seizures start to occur in the majority of rats at 3-4 weeks after SE [23]. Thus, due to the present experimental design at 7 days after induction SE by kainate it was not possible to state with certainty that the rats would develop spontaneous seizures. Nevertheless, although seizures were not yet present (apart from a few incidental observations), it can be assumed that at this time point after SE the brain is progressing towards the epileptic state. Furthermore, it is a common clinical observation that ^18^FDG uptake is decreased in epileptic brain tissue [54]. Actually, that is also what was found in this study: a lower ^18^FDG brain uptake in kainate compared to saline treated rats. Therefore, this indicates that at least some functional changes were brought about in the kainate treated rats that mimics what has been observed in the human epileptic brain.

Overall, parameter estimates were similar for individual and population modelling approaches. However, for the same model complexity, mixed effects modelling provided more precise parameter estimates. This is because in population modelling, the inter-animal variability is incorporated separately from the structural model parameters. A two (tissue) compartment brain model best described cerebral kinetics of *(R)*-[^11^C]verapamil. This is in line with previous reports using *(R)*-[^11^C]verapamil in combination with a P-gp inhibitor in rats [26,28,55].

Tariquidar treatment was incorporated as a categorical rather than continuous covariate in the population model. This was justified, as differences in plasma tariquidar concentrations were small and no relation between the brain-to-plasma concentration ratio, K_p, _and plasma tariquidar concentration was observed. Tariquidar treatment resulted in approximately an 11-fold increase in K_p_. This is in agreement with previous studies performed in naive (control) rats using either tariquidar or cyclosporine A as P-gp inhibitor [25-28].

Since P-gp is expressed at the BBB, an upregulation of P-gp function, as speculated in epilepsy, could either decrease the clearance into the brain, Q_in_, or increase the clearance out from the brain, Q_out_. However, inclusion of rat group (saline or kainate treated) as covariate on the parameter Q_in _(which is related to K_1_, the parameter usually used to describe the blood to brain transfer of PET ligands, as K_1 _= Q_in_/V_c _*V_c_/V_br1+2_) or on the parameter Q_out _did not improve the population model fit. Instead, inclusion of rat group as covariate for the brain distribution volume, V_br1, _did improve the model fit significantly and increased V_br1 _with 32% in kainate treated compared to saline treated rats. Therefore, these data do not provide evidence of increased P-gp expression or functionality at the BBB at the time point after kainate treatment chosen in this study. However, in the brain P-gp is not only expressed at the endothelial cells of the BBB but also at other cell types within the brain [56]. If the kainate treatment would indeed have resulted in increased P-gp expression on brain parenchymal cells, then inhibition of P-gp expressed in brain parenchymal cells in kainate treated rats could cause a larger intracellular distribution, compared to control rats. As a consequence a V_br1 _would increase. This was indeed found for *(R)*-[^11^C]verapamil in the brain of kainate treated rats in the present study: the increase in V_br1 _resulted in a slower elimination of *(R)*-[^11^C]verapamil from the brain. However, as PET cannot distinguish between extracellular and intracellular *(R)*-[^11^C]verapamil the PET data may only indicate increased intracellular distribution. Microdialysis would be the method of choice to investigate intra-brain distribution further. In this context, the findings of Bankstahl et al. are of interest, as they reported that after SE, total brain to plasma ratio of phenytoin in rats was lower, while the opposite was found in brain dialysate to plasma ratio [20]. As discussed previously *(R)*-[^11^C]verapamil is the most frequently used radiotracer for studying P-gp function with PET. Recently a new radiotracer, [N-methyl-^11^C]N-desmethyl-loperamide has been developed [57]. This radiotracer could potentially be better suited for studying P-gp function since it produces less radiolabelled metabolites compared to *(R)*-[^11^C]verapamil and hence gives a purer signal. The problem with a low baseline signal still remains with this novel tracer. Bartmann et al used the 5HT_1A _receptor ligand [^18^F]MPPF (fluorine-18-labeled4-(2;-methoxyphenyl)-1-[2;-(N-2"-pirydynyl)-p fluorobenzamido]ethylpiperazine) to study brain uptake in a chronic rat model of epilepsy and found that tariquidar treatment increased the transport into and decreased the transport out from the brain more in AED non-responders compared to AED responder rats [58]. This is in line with the notion that AED non-responders have an increased P-gp functionality compared to AED responders. The advantage of using a receptor ligand such as [^18^F]MPPF is that the baseline brain penetration is higher compared to *(R)*-[^11^C]verapamil and [N-methyl-^11^C]N-desmethyl-loperamide. However, binding to receptors within the brain might potentially confound the results.

Finally, changes in blood flow due to tariquidar treatment or disease state could potentially confound the results if brain distribution of *(R)*-[^11^C]verapamil would be limited by blood flow. There are, however, no studies indicating that tariquidar would alter blood flow and there are no studies comparing blood flow in kainate treated rats compared to controls.

## Conclusions

In tariquidar co-administered animals, a decrease in brain uptake of *(R)*-[^11^C]verapamil was observed in kainate treated compared to saline treated rats in an early phase of 7 days following induction of SE. However, the clearance out of the brain was also slower in the kainate treated rats, resulting in similar brain-to-blood ratios (K_p_) for both groups. No significant changes in P-gp expression were found. Further investigations have to show if P-gp expression/functionality is altered in a later stage of epilepsy development.

## Competing interests

The authors declare that they have no competing interests.

## Authors' contributions

SS, ECL and RAV planned the study. ADW and GL were responsible for radiochemistry and metabolite analysis, respectively. SS, GL and CFMM organized and performed the animal studies. MCH was responsible for the PET scanner. SS performed the data analysis with assistance from MCH and AAL. SS, ECL and RAV wrote the manuscript. All authors read and approved the final manuscript.

## Pre-publication history

The pre-publication history for this paper can be accessed here:

http://www.biomedcentral.com/1471-2342/11/1/prepub
